# Retraction Note to: A novel miRNA identified in GRSF1 complex drives the metastasis via the PIK3R3/AKT/NF-κB and TIMP3/MMP9 pathways in cervical cancer cells

**DOI:** 10.1038/s41419-025-07899-0

**Published:** 2025-07-28

**Authors:** Qi Sun, Zhen Yang, Pu Li, Xu Wang, Lu Sun, Shixing Wang, Min Liu, Hua Tang

**Affiliations:** 1 Tianjin Life Science Research Center and Department of Pathogen Biology, Collaborative Innovation Center of Tianjin for Medical Epigenetics, School of Basic Medical Sciences, Tianjin Medical University Tianjin, 300070 China; 2 Tianjin Central Hospital of Gynecology and Obstetrics, Tianjin, 300100 China

Retraction Note to: *Cell Death and Disease* 10.1038/s41419-019-1841-5, published online 02 September 2019

The Editor-in-Chief has retracted this article. After publication, a number of concerns were raised regarding the images in the article. Specifically:

- Figure 2G Anti-miR-G-10 appears to contain overlap between several panels
- Figure 4E HeLa pcDNA3 0h and pSilencer-NC 0h appear to overlap
- Figure 4E HeLa pcDNA3/TIMP3 0h and pSilencer-NC 0h appear to overlap
- Figure 4E C33A pcDNA3 48h and shR-TIMP3 0h appear to overlap
- Figure 6A pcDNA3 at 30 minutes, 60 minutes and 90 minutes, and miR-G-10 at 60 minutes and 90 minutes appear to overlap
- Figure 6A Anti-miR-G-10 at 10 minutes and pSilencer-NC at 30 minutes appear to overlap
- Four panels within figures 2F, 4C and 5C appear to overlap representing different conditions in each instance
- the DAPI panel in figure 7F pcDNA3 + pcDNA3, and miR-G-10 + shR-PIK3R3 appear to overlap with rotation

The authors did not respond to correspondence from the publisher regarding these concerns. The Editor has lost confidence is the data and conclusions of this article.

Author Hua Tang could not be contacted by the publisher. All other authors did not respond to the publisher regarding this retraction.

